# An improved method for undertaking limiting dilution assays for *in vitro *cloning of *Plasmodium falciparum *parasites

**DOI:** 10.1186/1475-2875-10-95

**Published:** 2011-04-18

**Authors:** Alice S Butterworth, Alan J Robertson, Mei-Fong Ho, Michelle L Gatton, James S McCarthy, Katharine R Trenholme

**Affiliations:** 1 Clinical Tropical Medicine Laboratory, Queensland Institute of Medical Research, Herston, Brisbane, 4006, Australia; 2 School of Medicine, University of Queensland, Herston, Brisbane, 4006, Australia; 3 Malaria Drug Resistance and Chemotherapy Laboratory, Queensland Institute of Medical Research, Herston, Brisbane, 4006, Australia; 4 Malaria Biology Laboratory, Queensland Institute of Medical Research, Herston, Brisbane, 4006, Australia

## Abstract

**Background:**

Obtaining single parasite clones is required for many techniques in malaria research. Cloning by limiting dilution using microscopy-based assessment for parasite growth is an arduous and labor-intensive process. An alternative method for the detection of parasite growth in limiting dilution assays is using a commercial ELISA histidine-rich protein II (HRP2) detection kit.

**Methods:**

Detection of parasite growth was undertaken using HRP2 ELISA and compared to thick film microscopy. An HRP2 protein standard was used to determine the detection threshold of the HRP2 ELISA assay, and a HRP2 release model was used to extrapolate the amount of parasite growth required for a positive result.

**Results:**

The HRP2 ELISA was more sensitive than microscopy for detecting parasite growth. The minimum level of HRP2 protein detection of the ELISA was 0.11ng/ml. Modeling of HRP2 release determined that 2,116 parasites are required to complete a full erythrocytic cycle to produce sufficient HRP2 to be detected by the ELISA. Under standard culture conditions this number of parasites is likely to be reached between 8 to 14 days of culture.

**Conclusions:**

This method provides an accurate and simple way for the detection of parasite growth in limiting dilution assays, reducing time and resources required in traditional methods. Furthermore the method uses spent culture media instead of the parasite-infected red blood cells, enabling culture to continue.

## Background

The use of single parasite clones is an essential part of many malaria experimental studies. Techniques requiring clones include: isolating parasite lines from field samples, genotyping, drug resistance testing and genetic manipulation [1-3]. These studies require single clonal parasite isolates to ensure accurate and unambiguous results.

Limiting dilution is the standard method for *in vitro *cloning of *Plasmodium falciparum *isolates. The method entails the culture of parasites in a 96-well microtiter plate at dilutions below one parasite per well for 2-4 weeks [4]. At the appropriate dilution, individual clones grow in a single well. Detecting parasite growth by microscopic examination of smears in such limiting dilution assays is time consuming and laborious, thereby restricting the concurrent analysis of multiple plates [4]. Another popular method is the lactate dehydrogenase assay (pLDH) [5]. While this technique is simple to use, it requires that parasitized red blood cells be tested, thereby further attenuating parasite culture. Other methods of detection that have been described include PCR and flow cytometery, both of which require specialist equipment and knowledge [6-8].

Histidine-rich protein II (HRP2) is a water-soluble non-labile protein produced by *P. falciparum *across the asexual cycle and is secreted into parasite culture media where it can be detected by an antigen capture ELISA. HRP2 detection by ELISA is a fast, accurate method, and only requires the use of spent culture media ensuring that culture can continue. In this experiment the detection of HRP2 by ELISA using a commercially available kit as a means to facilitate the limiting dilution assay was evaluated.

## Methods

### Materials

The following ELISA was used: Malaria Ag. Pf. ELISA (Standard Diagnostics Korea; product code 05EK50).

### Detection of parasites by HRP2 ELISA vs. microscopy

The *P. falciparum *strain 3D7 was subject to routine *in vitro *culture [9]. Asynchronous cultures were evaluated by microscopy to determine parasitaemia and to ensure they contained > 90% singly infected erythrocytes. Haematocrit was measured using a haemocytometer. A suspension was prepared that contained 50 parasitized erythrocytes at a haematocrit of 2% in 10 ml of culture media. This was plated out at 0.5 parasites per well in a 96 well sterile microtiter plate, adding 100 ul per well. Three wells of non-parasitized erythrocytes at 2% haematocrit were included as a negative control. Culture media was changed at days 4, 7, 11, 14 and 18 using a multichannel pipette. Parasite growth was detected by HRP2 Antigen (Ag) by ELISA (Standard Diagnostics Korea) using spent culture media on days 14 and 21. Assays were performed in accordance with the manufacturers instructions with one exception; 50 μl of spent media was added to 75 μl of conjugate, instead of 100 μl of sample to 150 μl of conjugate. All wells were assessed by microscope examination of thick smears by two operators. The data presented here is one representative experiment of three.

### HRP2 antigen quantification

Freshly prepared serial dilutions of 3D7 parasite culture supernatant taken from a frozen stock were used as control standards in each ELISA. The concentration of HRP2 in this culture supernatant had previously been measured at 55.5 ng/ml. This concentration had been determined by interpolating the ELISA optical density of serial dilutions of this culture supernatant against a stock of recombinant HRP2 protein with known protein concentration (Nelson Lee, manuscript in preparation). The HRP2 antigen level in ELISA assays was interpolated from a four parameter standard curve using the software package Softmax Pro (Molecular Devices Inc.). The cut-off for a positive result was set at the mean + 3 standard deviations above the red blood cell control sample optical densities. The threshold for HRP2 detection was determined in ng/ml and converted to ng/50 μl to reflect the volume of supernatant tested in the assay.

### HRP2 release model and minimum parasite numbers

The minimum parasite number required for detection was calculated by dividing the HRP2 antigen detection threshold of the ELISA by the amount of HRP2 produced per parasite per cycle [10]. A model predicting the accumulation and release of HRP2 in a well containing a single parasite over time was derived. The model was based on a replication rate of 2-10 replications per cycle with 5.2 fg of HRP2 secreted/parasite/erythrocytic cycle [10]. The following is the equation used for the model: HRP2 accumulation = 5.2 fg × (replication rate × parasite number of previous generation). Replications were defined as the number of parasites produced by one parasite during one cycle of asexual reproduction. The ELISA threshold of detection was doubled to reflect that only half the volume of the well is used in the ELISA and therefore half the accumulated HRP2 can be detected. This threshold was then applied to the model and the asexual cycle at which the threshold would be reached was obtained across a range of replication rates. The cycle at which the HRP2 threshold would be reached was then converted into days, assuming a duration of the asexual cycle of 48 hours, beginning at ring forms and ending at schizont rupture. As this conversion would be affected by using asynchronous cultures, the estimated day the threshold of HRP2 accumulation is reached is ± 12 hours the prediction. The model also assumed media changes every 48 hours, which is the length of an asexual cycle.

## Results

### ELISA vs. microscopy

To compare the sensitivity of the HRP2 antigen ELISA assay and microscopy, all plate wells were smeared and assessed. At day 14, 54% (49/91) of the wells were positive by ELISA, of which two were negative by microscopy (47/91). At Day 21 all wells positive by ELISA (49/91) were also positive by microscopy (49/91). All wells negative by ELISA were negative by microscopy.

### The ELISA HRP2 detection threshold and HRP2 release model

**Figure 1:**
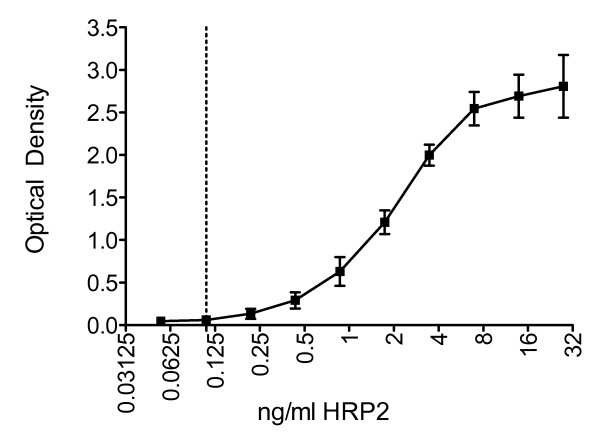
**HRP2 ELISA Detection Threshold**. Optical density (OD) vs. ng/ml of HRP2 in the HRP2 ELISA. Error bars represent the standard deviation of OD across the replicate assays. The HRP2 threshold of detection of the ELISA = 0.11 ng/ml.

The limit of sensitivity of the HRP2 ELISA kit was 0.11 ng/ml (Figure 1). This equates to 5.5 pg of HRP2 in 50 μl of culture supernatant used in the ELISA. As half the culture supernatant volume of each well in a dilution plate is used in the assay, 11 pg of HRP2 is required to accumulate in 100 μl of supernatant to reach a detectable level by ELISA. By applying previously published data indicating that 5.2 fg of HRP2 is released by one parasite per erythrocytic cycle [10] it follows that 2,116 parasites would be required to complete one erythrocytic cycle in order for HRP2 levels to accumulate to the detection threshold.

**Figure 2:**
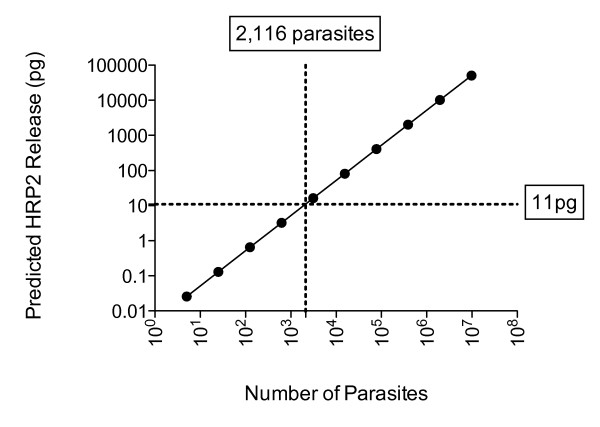
**HRP2 Release Model**. Predicted amount of HRP2 released during a limiting dilution experiment. The model is based on a starting point of 1 parasite per well replicating at a factor of 5 per 48-hour cycle. The points on the graph represent the predicted number of parasites and HRP2 release at the end of each 48-hour erythrocytic cycle. The dotted lines are the accumulation threshold for detection by ELISA (horizontal) and the corresponding number of parasites (vertical).

The time point when HRP2 antigen levels in the cultures reach the ELISA detection threshold depends on the parasite replication rate, as well as the timing of media changes. HRP2 accumulation has been shown to correlate directly with parasite growth rate [11]. The replication/growth rate of parasites depends on many factors, such as parasite strain and *in vitro *culture conditions. In order to determine when a well containing a single parasite at the start of an experiment would reach the HRP2 antigen detection threshold, a HRP2 release model was constructed. In this model the 5.2 fg HRP2 produced per cycle is used in an equation with the predicted exponential growth rate of between 2-10 replications per parasite per cycle. In figure 2, a parasite replication rate of 5 per cycle was applied to the sensitivity index. The model predicts that a single starting parasite will reach > 2,116 parasites after 5 cycles or 10 days of culture.

The model predicts that at a minimum growth rate of 3 replications per cycle, HRP2 accumulation will reach the point of detection of the ELISA by day 14 of culture. Applying a replication rate of 10 per cycle, the positive wells should be detectable in 8 days. All predictions of time to reach the detection threshold are, ± 12 hours if using an asynchronous culture. In the limiting dilution experiment reported here, all wells containing parasites were positive by day 14, as predicted by the model.

## Discussion

Cloning by limiting dilution is an indispensable method in malaria research. However, it is time consuming and labor intensive. The use of a commercial ELISA kit represents a timesaving alternative method for detection of *P. falciparum *growth in such experiments. In addition, it holds a distinct advantage over other methods, such as the pLDH assay, as the HRP2 antigen ELISA uses spent culture media, not parasitized erythrocytes, thereby ensuring that culture can continue.

Recently described methods of detection, PCR and flow cytometry, require specialist equipment and trained operators. The ELISA method is simple and quick to perform and does not require expensive reagents. The ease of this method ensues successive plates can be run, allowing a higher volume of plates to be analyzed at once. This is in contrast to the traditional method of microscopy, which is time consuming and relies on the skills of the operator. Furthermore, 2-week-old cultures traditionally produce smears that are difficult to read, containing a large amount of parasite debris. In this work, microscopy proved to be the least sensitive method. The HRP2 ELISA described is highly sensitive, detecting the presence of HRP2 produced by *P. falciparum *parasites in spent culture media at a limit of detection of 0.11 ng/ml.

The relationship between the ELISA detection threshold and number of parasites is difficult to directly measure as the method detects the amount of HRP2 antigen that accumulates over time and thereby depends on the parasite replication rate. The replication rate of a parasite depends on many variables, such as culture conditions, i.e. the availability of nutrients, temperature and gas composition; and parasite strain, as growth rates vary according to a parasites origin and the time a parasite has had to adapt to *in vitro *culture. The data reported here in conjunction with the HRP2 antigen modeling, indicates that undertaking the HRP2 Ag ELISA after 14 days of culture should result in the identification of all positive wells, even with parasite replication rates as low as 3 per cycle. As the method detects HRP2 that has accumulated, detection of clones also depends on the length of time since the media was changed prior to the assay. As 89% of HRP2 is released it the point of schizont rupture [10], allowing a minimum of 48 hours (which corresponds to one erythrocytic cycle) between media change and ELISA, ensures that the HRP2 will accumulate to detectable levels.

## Conclusions

Parasite detection by HRP2 ELISA is a sensitive and accurate way of overcoming the rate-limiting step of the limiting dilution method. Using ELISA allows large scale limiting dilution experiments to be performed simultaneously, thereby increasing productivity. Furthermore, the method allows culturing to continue after the assay, a distinct advantage over previously described methods.

## Competing interests

The authors declare that they have no competing interests.

## Authors' contributions

ASB performed the limiting dilution experiments, ELISA, microscopy, modeling and data analysis. AJR assisted with the ELISA methodology. MFH: defined the standard. MLG provided guidance for the HRP2 modeling. JMC supervised and assisted with methodology, modeling and analysis. KRT supervised and assisted with the limiting dilution experiments, methodology and analysis. All authors read and approved the final manuscript.
